# Obturation among the Ancient Greeks

**Published:** 1858-07

**Authors:** 


*Obturation among the Ancient Greeks.*—According to the Archives of Pharmacy, Lauderer has observed in a museum at Athens, in the upper jaw of an ancient cranium, a tooth filled with gold foil, perfectly pure, as an analysis determined. The skull was found in one of the tombs which date from the days of ancient Greece.—*Ibid.*

